# A national test of dyslexia

**DOI:** 10.1007/s11881-023-00285-5

**Published:** 2023-07-07

**Authors:** Mads Poulsen, Holger Juul, Carsten Elbro

**Affiliations:** Department of Nordic Studies and Linguistics, University of Copenhagen, Emil Holms Kanal 2, 2300 Copenhagen S, Denmark

**Keywords:** Dyslexia, Identification

## Abstract

Different definitions and tests of dyslexia can cause unfairness and make life difficult for people with dyslexia as well as for the professionals. In 2012, the Danish government decided to support the fight against dyslexia. The government issued a public tender for the development of “a standardized, electronically administered test of dyslexia for use […] from primary Grade 3 and up through all educational levels to 5-year university education.” The present paper reports from the development of this National Dyslexia Test. The paper focuses on the definition of dyslexia and the composition, reliability, and validity of the test. Data from the development of the test demonstrate the psychometric properties of the test. Reliability was indicated by a high agreement between the two (computer-administered) measures that are part of the test. External convergent validity was indicated by a high agreement between test results and results from prior practice and by agreement between test results and reading comprehension of educational texts. The paper concludes with a discussion of the practical uses and potential issues with the test since its release in 2015.

## Introduction

Difficulties with learning to read and write usually cause worries. Children wonder what is wrong with them; parents become seriously concerned about their children and their home environment. A dyslexia diagnosis can be a relief to both a child and their parents (Andreassen et al., 2006; Battistutta et al., 2018). A dyslexia diagnosis can also open doors. It may entail provision of special support, assistive technology, intervention, and remedial teaching with a high teacher-to-student ratio. It may lead to special concessions at exams and entry into further education. All of this depends on national and local legislation and practice.

Given the importance of a dyslexia diagnosis, dyslexia testing is high-stakes. It is impractical and confusing when several different tests are used concurrently, especially if they are based on different definitions of dyslexia. The potential benefits of a unified theoretical and operational definition can be many: a consensus across different parts of the educational system can promote transparency, consistency, and fairness and reduce the number of tests the individual would have to take when entering a new level of education or a new employment. The situation may be somewhat different between countries, but even in a very homogeneous society as the Danish, one could observe large local and regional differences in definition and support for people with dyslexia. Around 2002, several communal educational-psychological counseling offices did not acknowledge dyslexia at all, but only operated with the general term “reading disabilities” or “reading delays.” The number of support hours per student (across all students) in a commune ranged from approximately 0.2 to 6.9 per year, that is, from 2.1 to 86.6 h per student per year at a prevalence of 8% (Haven & Nielsen, 2004). Given the scarcity of public communal support, many parents of children with dyslexia had no other choice than to refer to private consultants who would apply their own definitions and cures without adherence to public guidelines or educational requirements. With no agreed-upon definition of dyslexia, students with dyslexia were also required to undergo new testing and diagnosis whenever they crossed administrative boundaries between age groups, educational levels, and institutions.

In 2012, the Danish Parliament decided that the situation was unacceptable and the Ministry for Education issued a public tender for the development of a National Dyslexia Test. “The task is to develop a standardized, electronically administered test of dyslexia for use […] from primary Grade 3 and up through all educational levels to long university education. […] The aim is to have a standardized tool to serve a uniform identification of dyslexia in order to secure targeted guidance, teaching, and support for students with dyslexia. […] Uniform identification is of particular importance at transitions within and between educations” (Danish Ministry of Education, 2012).

A good dyslexia test does not override political decisions. Different districts and states may have their own policies about both cut-offs on the dyslexia scale and different provisions for the dyslexic individual. For example, the Danish Ministry of Education decided to adapt an *economically neutral* cut-off on the dyslexia scale, i.e., to set a cut-off that resulted in the same absolute number of students with a dyslexia diagnosis before and after the introduction of the National Dyslexia Test. This political decision does not guarantee that everyone who may benefit from support receives it. However, to the best of our knowledge, there is no way to determine a true cut-off point or prevalence (cf. Wagner et al., 2020). For example, there is no golden standard for determining the severity of everyday problems with written communication that would define a reading difficulty.

The National Dyslexia Test was introduced in February 2015. Since then, the test has been optional and only to be taken when indicated by, e.g., dyslexia among close relatives, difficulties in the development of precursors of reading and spelling, and seriously delayed reading and spelling development in the first grades. However, parents can demand that their children are tested at least once. The National Dyslexia Test is only to be administered by qualified reading specialists who can conduct testing reliably and validate test results against other available information about the student’s problems.

Of course, intervention against dyslexia can and should be initiated long before the end of Grade 3 following a positive result on the National Dyslexia Test (Poskiparta et al., 2003; Torgesen, 2005). In the early school years, an optional *At-risk Test of Dyslexia* is available (Danish Ministry of Education, 2022, based on Gellert & Elbro, 2018). It is not a test of dyslexia, but of increased *risk* of dyslexia, because it is too early to diagnose dyslexia before a child has had a proper chance to learn to read with high-quality, targeted instruction provided by a reading specialist (as in the US tier system).

The present paper reports on the structure of the National Dyslexia Test, its reliability, and validity and discusses a few aspects of its uses since its introduction.

### A simple, operational definition of dyslexia

The National Test of Dyslexia is based on the following definition: *dyslexia is marked difficulties with learning to read and write caused by slow and/or inaccurate mapping of letters and letter sequences onto sounds* (Danish Ministry of Education, 2012). This definition is closely related to the core of the current definition by the International Dyslexia Association (IDA, Lyon et al., 2003).

Like the IDA definition, the official Danish definition is based on a distinction between decoding and other component processes of reading. Dyslexia is specific to difficulties with the acquisition of decoding in reading and distinct from specific difficulties with language comprehension in reading (as implied by “The Simple View of Reading,” Hoover & Gough, 1990). This means that dyslexia may occur in combination with difficulties with specific language comprehension in the case of mixed (“garden variety”) reading difficulties.

There are two reasons for the sharpened focus on the acquisition of *the ability to use* the alphabetic principle (the ability to map letters to sounds) in the National Test definition than in the IDA definition. Firstly, this focus links dyslexia to the ability to use the core principle of any alphabetic orthography. By doing so, it makes it clear why dyslexia is such a *fundamental problem* in learning to read and write without defining dyslexia on the basis of an underlying condition (Catts & Petscher, 2021). It also distinguishes between dyslexia and individual *compensation* by means of other strategies in word reading, such as morphological analysis (van Viersen et al., 2019). Secondly, word knowledge (vocabulary) influences real-word recognition. It is easier to read known words than unknown words. This causes tests of real-word recognition to be biased against second language (L2) readers and others with a limited exposure to the language of the test. Such a bias can lead to an overdiagnosis of dyslexia in L2 readers and others (Elbro et al., 2012). The National Test definition reduces this risk by specifying a less language-specific core problem.

From the viewpoint of the IDA definition, the National Test definition of dyslexia may appear to overlook specific problems with whole-word recognition (with the “direct route” in dual-route models, Ottosen et al., 2022). However, ability to use the alphabetic principle is necessary not only for reading novel words but also for the development of orthographic whole word recognition. Letter-sound associations are the “glue” that ties orthographic representations of whole (sight)words to their phonological representations in the mental lexicon of the reader (Ehri, 2020). Hence, even though some dyslexic readers manage to learn to recognize some words as wholes, they are prone to making word-reading mistakes because the written words are only partially anchored to the spoken words. If the alphabetic principle is fully mastered, specific problems with the development of whole-word recognition are very rare even in deep orthographies as the English (Stanovich et al., 1997).

### The present study

The main purpose of the study was to assess the reliability and convergent validity of the National Dyslexia Test. Reliability was assessed by means of comparisons between the two (computer-administered) measures that form the National Dyslexia Test. Convergent validity was addressed by means of a comparison between the National Dyslexia Test results and prior practice, i.e., earlier referrals to special provisions because of reading and spelling difficulties. Convergent validity was also studied in a comparison between test scores and reading comprehension of educational texts. A high convergent validity is also indicative of a high reliability. A special concern was whether the same test could be used at all educational levels from Grade 3 and upwards through to long (5 + year) university education. In order to meet this concern, the study was carried out with a broad representation of students (at 10 levels) across the Danish educational system. Since the study was conducted before the test was made public, we could compare the results of the National Dyslexia Test with previous practice, i.e., whether or not participants were already provided special services because of reading difficulties. This comparison would not have been available had the study been carried out at a later point. A detailed description of the construction of the test may be found in the technical report by Møller et al. (2014).

## Methods

### Participants

The participants for the study were 1564 students from elementary Grade 3 through to long (5 + year) university education. The students were recruited from 10 different educational levels at three strata:

- Compulsory schooling: elementary and secondary Grades 3, 5, 7, and 9
- Upper secondary (college) education: vocational training, technical/commercial college, and general college (UK: A-levels, USA: advanced placement courses)
- Higher education: short (1–2 years, e.g., IT professionals), medium long (3–4 years, e.g., teacher, BSc engineering), and long (5 + years, e.g., MSc economics, medical doctor)

At each of these 10 educational levels, samples of about 100 students were recruited so as to be representative at the national level. For example, we sampled classrooms from primary and secondary schools with Grade 9 grade point averages at or close to the national average. Although this procedure is aimed at reflecting the national average, it may underestimate variation. At each of these 10 educational levels, some students received some kind of special support because of reading and spelling difficulties (“special service”). The two first columns of Table 1 provide an overview of the number of students in typical and special services in the (presumed) representative samples (and also show the percentage of students receiving special service, which provides an estimate of the prevalence of students receiving special service.).

**Table 1:** Sample characteristics

	Representative sample		Oversampled		
Educational level	Typical service *N*	Special service *N* (%)	Special service *N*	Total *N*	DL2 (%)
Compulsory schooling					
Grade 3	90	16 (15.1)	35	141	16.5
Grade 5	209	20 (8.7)	33	262	16.3
Grade 7	86	5 (5.5)	33	124	17.7
Grade 9	103	7 (6.4)	37	147	23.8
Upper secondary education					
Vocational training	134	12 (8.2)	34	180	13.9
Tech/commercial college	107	2 (1.8)	21	130	9.2
General college (A-levels)	108	6 (5.3)	42	156	12.8
Higher education					
Short higher ed. (1–2 y)	110	7 (6.0)	5	122	23.3
Medium higher ed. (3–4 y)	110	2 (1.8)	23	135	19.3
Long higher ed. (5 + y)	129	1 (0.8)	37	167	7.2

% = percentage of representative sample receiving special service. Oversampled = additional participants receiving special service. DL2 = percentage of total sample with Danish as a second language

In addition and to strengthen the database for the analyses of concordance with previous practice, we oversampled 20–40 students who were receiving special service in each of the ten educational groups. Table 1 columns 3–5 display the number of oversampled students, the total number of students, and the percentage of the total number of students for whom Danish was a second language.

The participants were recruited through contacts in municipalities or at their educational institutions. Oversampled students were recruited with the help of reading or education advisors at their institutions. All students participated after informed, written consent (parents gave consent in the case of minors).

### Measures

The National Dyslexia Test is a web-based assessment consisting of two subtests of mastery of the basic alphabetic code and a brief test of basic vocabulary knowledge. The test is self-contained with recorded spoken instructions and interactive practice items. Reading of real words was not included because of individual differences in word knowledge that were irrelevant to the mastery of the basic alphabetic code.

#### Pseudo-homophone identification

**Fig. 1:**
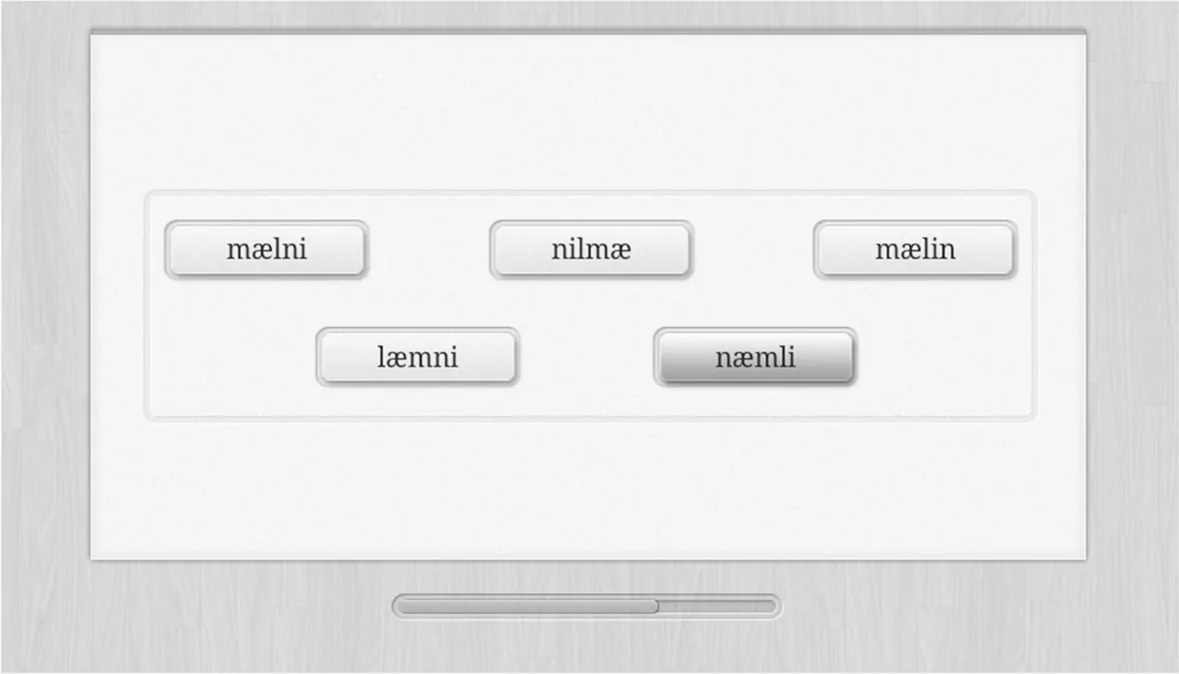
Pseudo-homophone identification. *Note*. The pseudo-word “næmli” is the correct choice because it is the only one that can be pronounced as a real word, *nemlig*, “namely,” in Danish

Each item in this multiple-choice task has five orthographic non-words among which one is a homophone to a real word (Fig. 1). In English, which one can sound like a real word: *tro*, *traf*, *gre*, *thaf*, *troo*? The expected answer is *troo* because standard letter sounds may provide the real word *true*. The task is introduced with spoken instruction and two trial items with corrective feedback. In the corrective feedback, the correct answer is pointed out on the screen and a spoken instruction explains how it sounds like a real word and the word is exemplified in a sentence context. It is pointed out how each of the distractors does not sound like a real word. At the end of the instruction, there is an option to restart the instructions. After instructions, participants have7 min to complete as many items as possible with a maximum of 44 items. There is no corrective feedback. The number of correct answers is corrected for guessing by subtracting the number of errors divided by 4; resulting negative scores are adjusted to zero (following Thorndike & Thorndike-Christ, 2009). The score is the number of correct answers (corrected for guessing) per minute where the latency is the median response time across items.

Previous studies have shown that measures of pseudo-homophone identification are highly correlated with other measures of “phonological coding in reading,” i.e., ability to use grapheme-phoneme mappings to read novel words (*r* = .82 in Elbro et al., 1994). A pilot test before the present study found a correlation of .72 between the pseudo-homophone task and an individually administered task of oral non-word reading.

#### Spelling of spoken non-words

**Fig. 2:**
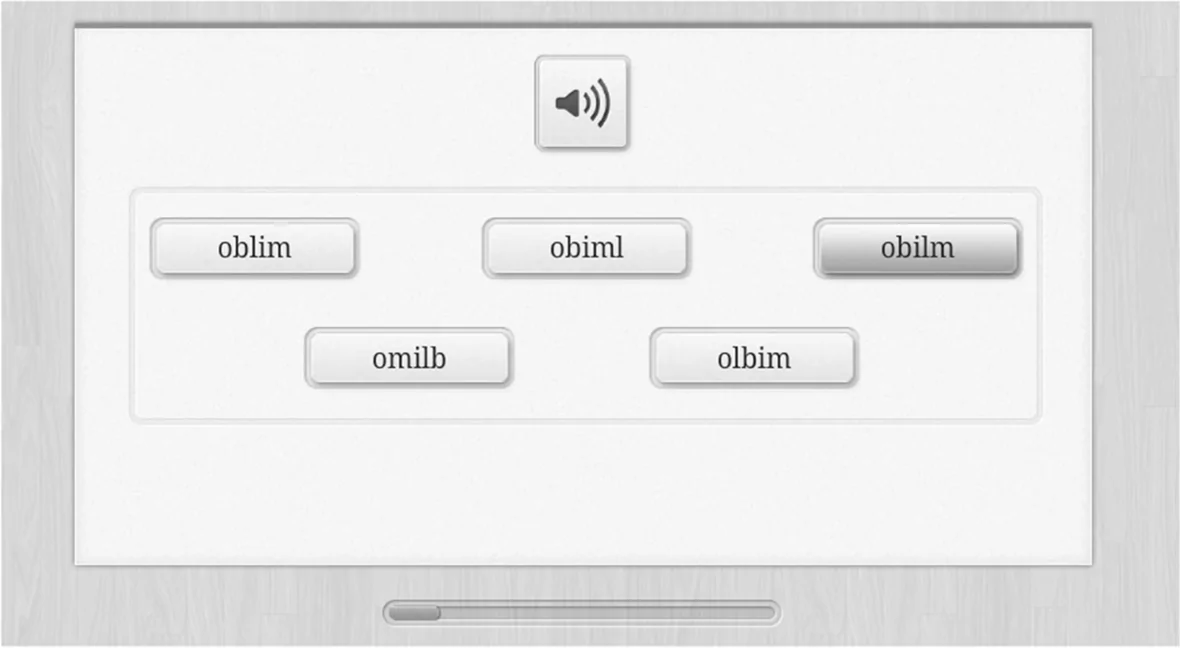
Non-word spelling. *Note. Obilm* corresponds to the spoken non-word

In this task, the participant hears a non-word and is asked to choose the appropriate spelling among five non-word possibilities (“find the right spelling”) (see Fig. 2). The participant can hear the non-word again by pressing the speaker button again. The multiple-choice format was chosen to eliminate differences in typing skills and familiarity with keyboards. The test is introduced with a spoken instruction and two trial items are provided with corrective feedback. During the corrective feedback, the correct answer is pointed out on the screen, a spoken instruction sound out the letters of the correct answer, and it is pointed out that the other options do not correspond to the non-word, which is repeated. At the end of the instruction, there is an option to restart the instructions. After the instruction, participants have 5 min to complete as many items as possible for a maximum of 40 items. The score is the number of correct answers (corrected for guessing) per minute (based on the median response time to all items). A pilot test found a correlation with oral non-word reading of .76.

#### Basic vocabulary

A measure of vocabulary knowledge was added to verify that problems with the pseudo-homophone task was not caused by a very limited knowledge of Danish words. Obviously, one needs to know that *nemlig* (“namely”) is a Danish word to be able to choose the right pseudo-homophone (*næmli*) (Fig. 1). Words for the vocabulary measure were chosen to match the words in the pseudo-homophone task in terms of frequency. In the vocabulary task, participants hear a spoken word and are asked to select the matching picture out of a selection of four. In order to avoid confusion, all target words and distractors are concrete nouns. Following spoken instruction and two trial items with corrective feedback, participants have 2 min to complete as many items as possible out of 15 items. This timeframe allowed most students to complete all items (the average number of items attempted was above 14.9 in all groups). The score taken from this measure is the number of correct items.

#### The National Dyslexia Test score

For ease of interpretation, an individual National Dyslexia Test score is calculated as the average scaled scores on each subtest: pseudo-homophone identification and non-word spelling. Scaling was done based on Grade 9 means and standard deviations and transformed to a scale with a mean of 100 and a standard deviation of 15. Grade 9 represents the endpoint of compulsory education, and general progress on the dyslexia scale was modest beyond Grade 9 (see “Results” below). The test report generated for practitioners includes the National Dyslexia Test score and, in addition, standardized subtest scores and raw accuracy and efficiency scores for the two subtests to afford some insight into individual difficulties with accuracy and/or speed. Below, we report only raw scores for subtests.

As mentioned in “Introduction,” The Danish Ministry of Education decided to make the new dyslexia test and referral practice economically neutral. This meant that a cut-off is set on the National Dyslexia Test scale at the 8th percentile because on average about 8% of students were already receiving some kind of special service because of reading difficulties. Based on the results from the present study, separate cut-off values corresponding to the 8th percentile were computed at each grade level from 3 to 9. All educational levels after Grade 9 shared the same cut-off values corresponding to the cut-off value in Grade 9. Cut-off values were based only on the representative samples with the consequence that cut-off values were based on relatively few observations. To compensate for this, cut-off values were estimated from the normal distribution using the observed means and standard deviations as parameters. The distributions were close to normal. It should be noted that most of the analyses in the “Results” section (e.g., correlational and area-under-the-curve analyses are independent of specific cut-off decisions).

#### Reading comprehension of educational texts

A test of reading comprehension of educational texts was devised in order to further validate the National Dyslexia Test scores. The main aim was to investigate whether students who scored low on the National Dyslexia Test also displayed difficulties reading their educational texts. Because dyslexia is only one type of reading difficulty, we did not expect that a low score on the dyslexia test would explain all cases of reading comprehension difficulties. A secondary aim was to examine whether possible difficulties in language comprehension might add to the explanation of reading comprehension difficulties with educational texts—as predicted by “The Simple View of Reading” (Hoover & Gough, 1990).

Because of resource limitations, this validation was only carried out among students in vocational training, more specifically, among students attending an introductory course at business schools (“merkantil grunduddannelse”). This introductory course qualifies for jobs as assistants in customer service, administration, commerce, etc. Vocational training was selected because comprehension of educational texts in vocational training has been known to be a problem for many years (Grønborg et al., 1994). A relatively high incidence of dyslexia might be one non-exclusive explanation which has not been investigated so far. Following advice from teachers at the participating schools, four widely used textbooks were selected, two on social studies and two on business economics. These textbooks were aimed at relevant subjects and levels but not part of the curriculum of the participating schools. In order to assess and interpret the difficulty of the selected texts for individual students, a cloze procedure was adopted following the method of Bormuth (1971, 1975). With this procedure, sections of text are selected systematically from each book and every 5th word replaced by underscores. Participants are then asked to infer or guess the original words and write them down on blank lines in the texts. This procedure has been validated against more traditional comprehension questions with educational texts, and cloze score levels have been aligned with measures from traditional question-answering tests (see “Results” below). The total number of words from each of the four textbook was about 420, and the total number of missing words to be replaced by the participants was 159. Participants were allowed a maximum of 2 × 12 min to complete as many items as possible from the texts on the two subjects of study. The score was percentage correct of the items attempted by the participant.

### Procedure

The National Dyslexia Test was administered at the participants’ educational institutions by trained assistants. Students participated in groups of 10–15 students in primary/secondary school and in intact classes at higher educational levels, typically comprising 15–25 students. Each participant was seated with their own computer and headphones.

## Results

Table 2 presents the descriptive statistics of raw scores from the pseudo-homophone identification, non-word spelling, basic vocabulary, and the correlations between the first two measures by educational level.

**Table 2:** Descriptive statistics of raw scores with correlations between the two measures of ability to use the alphabetic principle

Educational level	Pseudo-homophones		Non-word spelling		Correlations	Basic vocabulary	
	*M*	SD	*M*	SD	*r*	*M*	SD
Compulsory schooling							
Grade 3	4.47	2.38	7.22	2.84	.69	13.04	1.49
Grade 5	5.96	2.79	9.41	2.88	.68	13.80	1.39
Grade 7	7.00	2.78	11.02	2.96	.67	14.04	1.26
Grade 9	8.31	3.21	11.72	2.84	.68	14.28	1.13
Upper secondary education							
Vocational training	6.26	3.06	10.32	3.44	.76	14.14	1.68
Tech/commercial college	7.94	2.67	12.80	2.65	.73	14.64	0.94
General college (A-levels)	8.43	2.84	12.39	3.19	.74	14.45	1.13
Higher education							
Short higher ed. (1–2 y)	7.75	3.32	11.42	3.32	.74	14.41	1.47
Medium higher ed. (3–4 y)	8.27	3.03	12.09	3.11	.69	14.33	1.52
Long higher ed. (5 + y)	9.80	3.17	12.69	2.67	.56	14.78	0.83

As seen in Table 2, both subtests of ability to use the alphabetic principle showed a steady progress up through grade levels 3–9. Beyond Grade 9, scores depended on the chosen education. General and technical/commercial college students displayed higher averages than Grade 9 students while students in vocational training had lower averages. This pattern is likely to reflect self-selection into education based on social background and academic abilities (including reading). Such a trend would concur with the fact that a relatively high proportion of students in vocational training received special reading services (8% compared to 5% and 2% in other types of upper secondary education, cf. Table 1).

Regarding the reliability of the dyslexia test, the correlations between the two subtests were generally high (*r* > .67, corresponding to > .80 when Spearman-Brown corrected) with the exception of long higher education (*r* = .56). The relatively low correlation at this level was caused by lower correlations at the top end of the scales. This was to be expected since the tests were not designed to be sensitive to variation among very proficient readers. This aspect of the test stresses the importance of the validity of the test (below).

As intended, the basic vocabulary measure displayed a strong ceiling effect with all group means above 13 (of 15) correct. To test whether basic vocabulary played a role in pseudo-homophone identification, we conducted separate correlation analyses between the two measures in each educational group. *R*^*2*^ values ranged between .001 (long higher education) and .07 (short higher education) with a mean of .02. These low correlations suggest that virtually no students were limited by an insufficient basic vocabulary knowledge in their performance on the pseudo-homophone identification task.

### Comparison with the pre-existing classification

The first and most central validation of the National Dyslexia Test concerned its concordance with pre-existing referrals to special reading services.

**Fig. 3:**
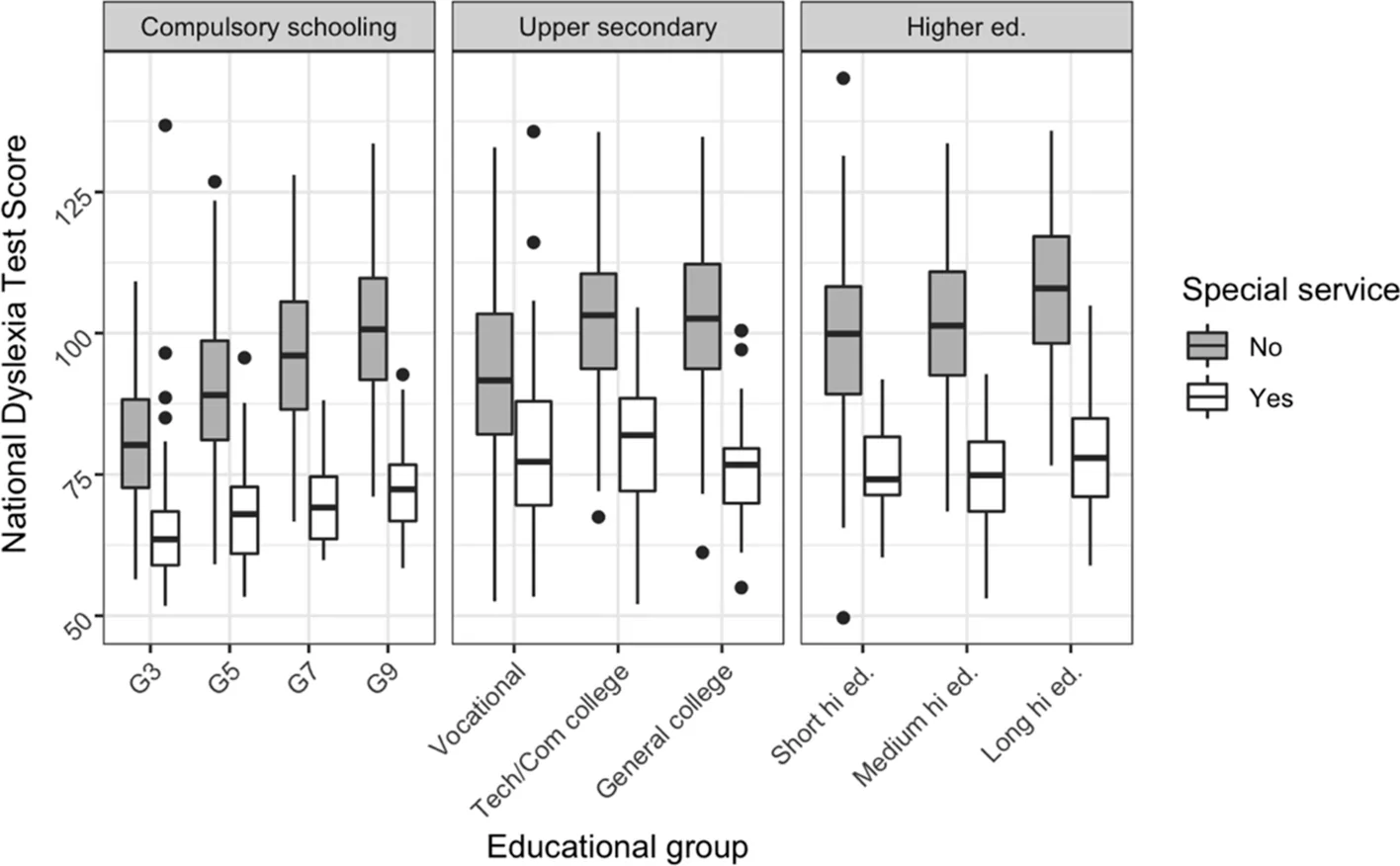
Boxplots of National Dyslexia Test scores by educational level in students who received special reading service vs. students who did not

Figure 3 presents boxplots comparing National Dyslexia Test scores in students who did or did not receive special reading services within each educational group. The figure includes data from the oversampled students.

The plots in Fig. 3 indicate good separation, except perhaps for students in vocational education and possibly for students in Grade 3.

Area under the curve (AUC) statistics were computed as a formal test of the concordance between the pre-existing classification and the National Dyslexia Test scores. The AUC provides a measure of how accurately the test score identify pre-existing classification by plotting the true-positive rate (sensitivity) against the false-positive rate (1 − specificity) across all cut-off values (Metz, 1978; Youngstrom, 2013). This statistic is used as a central criterion in evaluating psychological tests in general (Youngstrom, 2013) and recent evaluations of tests to identify language and reading disabilities in particular (e.g., Adlof et al., 2017; Compton et al., 2010; Nergård-Nilssen & Friborg, 2022). Table 3 displays the AUC values for the National Dyslexia Test in each educational group. Sensitivities and specificities for the official cut-off values (cf. above) are also displayed. AUC values were generally excellent (> .90) with possible exceptions of Grade 3 (AUC = .85) and students in vocational training (AUC = .75). The classification accuracy in long further education was excellent (AUC = .96) despite the low correlation between the two subtests in this group (Table 2 above). This supports the interpretation that the low correlation between subtests in this group was mainly due to divergence between the scales at the top end, which is unimportant for classification at the low end.

**Table 3:** Match between measured National Dyslexia Test scores and pre-existing classification

Educational level	AUC [95% CI]	Sensitivity	Specificity
Compulsory schooling			
Grade 3	.85 [.77–.92]	.43	.96
Grade 5	.93 [.89–.97]	.91	.82
Grade 7	.97 [.94–.99]	.84	.95
Grade 9	.97 [.95–.99]	.89	.93
Upper secondary education			
Vocational training	.76 [.68–.84]	.65	.74
Tech/commercial college	.89 [.82–.95]	.52	.93
General college (A-levels)	.95 [.91–.98]	.83	.93
Higher education			
Short higher ed. (1–2 y)	.90 [.82–.97]	.75	.88
Medium higher ed. (3–4 y)	.94 [.90–.98]	.80	.87
Long higher ed. (5 + y)	.96 [.93–.99]	.71	.96

*AUC* area under the curve statistics. Sensitivities and specificities are computed for estimated 8th percentile cut-off values

### Comparisons with reading comprehension of educational texts

A second validation was conducted with students in vocational training. The question was whether students with low scores on the National Dyslexia Test were at risk of difficulties understanding their educational texts. Since difficulties in reading comprehension may be caused not only by dyslexia but also by specific difficulties with language comprehension (or mixed difficulties), the validation also explored limited vocabulary knowledge as a potential source of text comprehension difficulties.

In order to carry out this validation, 105 of the 180 students in vocational training were recruited from four schools of commercial studies where they attended introductory courses (“merkantil grunduddannelse”). Complete datasets were obtained from 98 of these students; a few refused to share their data and some students only attended one of the two test sessions. Twenty-three of the students were receiving reading support. As mentioned earlier, these students were oversampled to strengthen the database.

The students in vocational training performed at relatively low levels on the National Dyslexia Test (Table 4). Their mean score of 89.7 was below that of Grade 9 students and the lowest of the mean scores at post-compulsory educational levels (cf. Figure 3). The students’ reading comprehension scores with educational texts were very low, averaging 29.4%. Following Bormuth (1971), scores below 35% indicate that the text is unsuitable for educational purposes. The 35% cut-off is based on comparisons with performance with ordinary comprehension questions and has been further validated in Danish (Nordentoft, 1981).

**Table 4:** Zero-order correlations between measures with descriptives added in the last line

	Dyslexia test score	Basic vocabulary	Reading comprehension
National Dyslexia Test score			
Basic vocabulary	.14		
Reading comprehension	.57**	.53**	
*M* (SD)	89.7 (15.5)	14.2 (1.6)	29.4% (11.5%)

Students in vocational training only (*N* = 98)

^**^p < .01

**Fig. 4:**
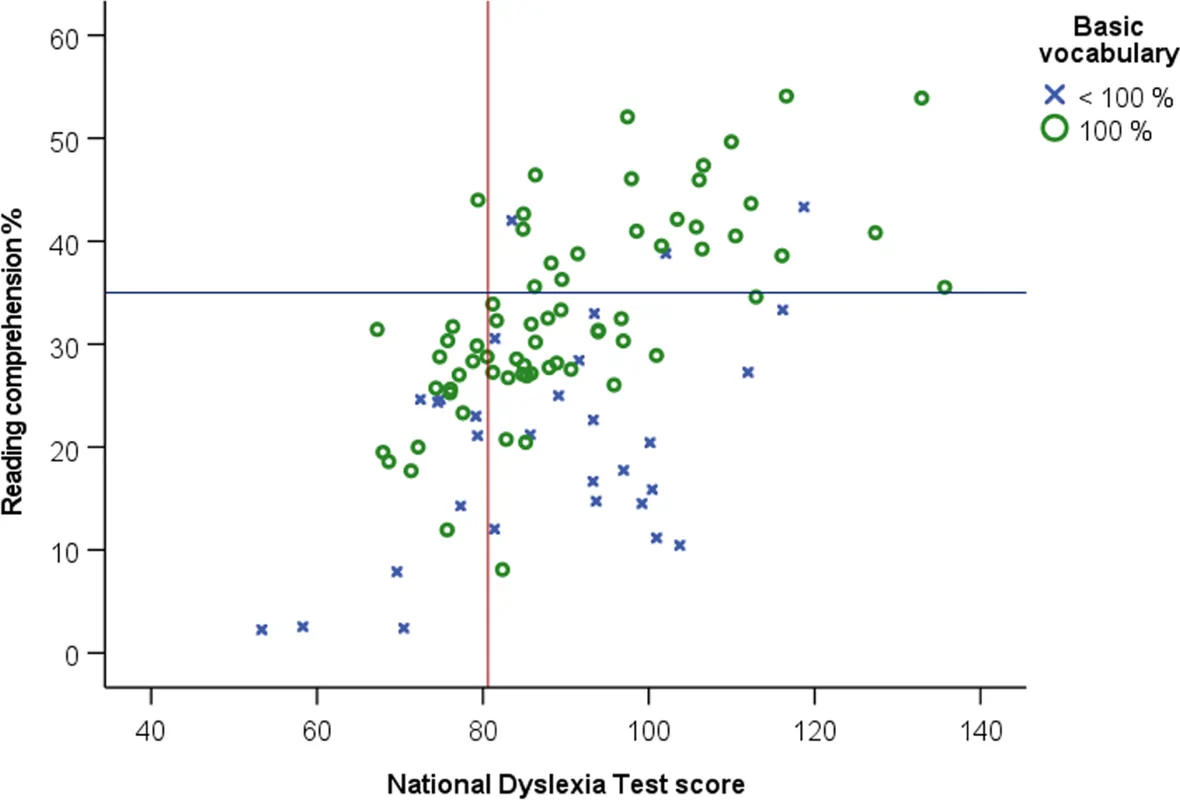
Reading comprehension plotted against the National Dyslexia Test scores and basic vocabulary scores. *Note*. The vertical line indicates the dyslexia threshold in post-compulsory education, and cut-off for comprehension difficulties is set at 35% (horizontal line). *N* = 98 students in vocational training

A closer look at the actual scores (Fig. 4) indicated that with just one exception, all students who scored below the dyslexia cut-off (vertical line) on the National Dyslexia Test had problems (scores below 35%) with reading comprehension of educational texts within their chosen subjects. These students were certainly not the only ones to experience reading comprehension difficulties. However, it may also be seen in Fig. 4 that many of the poorest comprehenders scored less than perfect on the basic vocabulary test. This is consistent with “The Simple View of Reading” (Hoover & Gough, 1990) that there is more than decoding (and dyslexia) to problems with reading comprehension.

In order to quantify the contributions of the National Dyslexia Test scores and basic vocabulary to reading comprehension, a linear regression analysis was conducted. The dependent variable was reading comprehension; the independent variables were the National Dyslexia Test score and basic vocabulary. The regression analysis indicated that both of the independent variables made substantial and significant contributions to individual differences in reading comprehension (dyslexia test score *β* = .54, *t* = 8.0, *p* < .001; basic vocabulary *β* = .50, *t* = 7.3, *p* < .001; total linear regression: *R* = .75, adjusted *R*^2^ = .56).

## Discussion

The results of the study suggest that the National Dyslexia Test is reliable and valid. The two separate measures of the ability to use the alphabetic code were found to be highly correlated at all 10 educational levels indicating convergent test reliability. The progression in student abilities was clear from Grade 3 through Grade 9, after which differences between educational groups appeared to mirror self-selection in the face of different educational demands for reading. When the two measures of the ability to use the alphabetic code were combined into one measure—the National Dyslexia Test score—they gave a very close match to pre-existing referral practice into special service in all educational groups, with possible exceptions at Grade 3 and among students in vocational training. These results attest to the convergent validity of the test. In addition, the National Dyslexia test explained a large proportion of the difficulties in reading comprehension among students in vocational training—an educational level that is known to attract a relatively high number of dyslexic students and students with the language of instruction (Danish) as a second language.

The match of the National Dyslexia Test to existing special provisions was less than excellent in two educational groups: Grade 3 and vocational education students. However, in both of these groups, the correlations between the two subtests were high (*r* = .69 and .76, respectively) suggesting acceptable reliability. In addition, the Grade 3 sample exhibited a noticeable higher proportion of referrals to special service (15% compared to 5–9% in Grades 5–9). The high referral rate may reflect a “better safe than sorry” approach based on multiple factors rather than specific difficulties with decoding. Thus, the National Dyslexia Test may provide a more stringent criterion for referral to special service. However, because of the relatively low sensitivity in Grade 3, the Ministry of Education currently encourages consideration of possible dyslexia in Grade 3 students in an interval *above* the 8% cut-off point. In vocational training, students may be more likely to experience other serious problems in addition to possible dyslexia than are students at other educational levels. The present study indicated that many students in vocational training may have limited reading comprehension because of problems with even basic vocabulary of the language of instruction. Such a mix of problems (mixed or “garden variety” reading difficulties) will inevitably lower the concordance between National Dyslexia Test results and earlier referral practice. There is certainly more to reading comprehension difficulties than decoding problems.

### Limitations of the study

The aim of the present study was not to investigate whether the National Dyslexia Test has, in fact, helped to “secure targeted guidance, teaching, and support for students with dyslexia.” There are good reasons to believe that it has—and has had—these functions, as the Danish government has strengthened legislation, for example, by requiring schools to provide the test at the parents’ wish as one of a number of governmental support initiatives (“dyslexia packages”) that have been implemented. A study of the impact of these initiatives is beyond the scope of the present paper though.

Among the more specific limitations of the current study, some deserves to be brought forward. The measure of basic vocabulary was never intended as a proper vocabulary measure not to mention a measure of language comprehension; it was just a control measure to make sure that participants have sufficient word knowledge to solve the pseudo-homophone task. Nevertheless, this very basic measure did account for substantial individual variation in reading comprehension after controlling for the National Dyslexia Test score. It seems reasonable to assume that the true influence of vocabulary (and language comprehension) on reading comprehension was underestimated in this part of the study.

Comparisons between the National Dyslexia Test scores and oral reading of non-words were only carried out in a smaller pilot test in two subgroups. This means that the findings from this earlier pilot study (high correlations) and the conclusions (a high convergent validity) should be interpreted with caution.

### Limitations of the National Dyslexia Test

This final part of the discussion touches on some challenges that have been observed by the Ministry and the authors mostly after the National Dyslexia Test was taken into practice. They were not part of the study of the test properties but are added here as potential targets for future research and practice.

As detailed in “Introduction,” *dyslexia does not cover all reading difficulties*. Unfortunately, it is common among non-professionals to call almost any developmental reading problem “dyslexia.” This popular view of dyslexia may present a problem to the face validity of a more well-defined test such as the National Dyslexia Test. However, to call many different reading problems “dyslexia” is a recipe for confusion, non-specific individual support, and unfairness. A relatively sharp definition of dyslexia will leave more space for consistent and educationally helpful definitions of other types of reading disabilities, such as specific problems with language comprehension in reading, reading in a second language, or lack of knowledge and practice with specific text genres. Yet, as long as these other problems are neither acknowledged, diagnosed, or supported economically and educationally, there will be continued pressure to widen the definition of dyslexia. However, one first step ahead could be to grant support towards more broadly defined disabilities that limit the educational potential of the student (as is current practice in Denmark). Specific diagnoses, such as dyslexia, may then be applied to target individual needs within this broader economic and educational framework.

*The same cut-off at all post-compulsory educational levels?* The cut-off points on the National Dyslexia Test scale are results of political decisions and they can certainly be discussed. For example, the same cut-off is applied to all students beyond compulsory schooling irrespective of the rather different demands for literacy at different educational levels. It is a fair but complex question whether such a one-limit-fits-all practice is educationally reasonable and economically sound. The demands for reading abilities are obviously higher in many university degree courses than in most vocational training. The present Danish policy is to encourage consideration of possible dyslexia even in university students who score within a specific interval above the Grade 9 cut-off. However, this is a complex issue because a great many factors may be taken into consideration.

*Control for individual practice?* Another limitation resides with the poor control for individual practice. All current definitions posit that dyslexia is a learning disability; that is, the learning outcome is much smaller than would be expected from the efforts of the student. With the National Dyslexia Test, as with practically all other dyslexia tests (though see Elbro et al., 2012), only grade (and educational) level is applied as a measure of the amount of effort that the learner has invested. This clear limitation is obvious in the case of students who make a special effort to learn to use the orthographic code, for example, in the extreme case, through studies of phonetics and phonology on their way to become speech and language therapists. We have evidence of students with this profile at our department though their prevalence is unknown. Such “practiced” students may pass the National Dyslexia Test because they have reached a basic level of ability with letter-sound mappings, but they may still be slow at getting any better at using these mappings and at learning the spelling of new words. Very practiced dyslexic students are not served well by the current cut-offs. There may be good reasons to continue to provide support for these students.

*How long should a dyslexia diagnosis be valid for?* A main point of the National Dyslexia Test is to reduce the number of times a student will have to take a test of dyslexia, especially when moving from one education (or level) to another. The fewer the times, the lesser are the concerns and worries about the continued access to support and special conditions. Currently, a positive result on the National Dyslexia Test is currently valid for 15 years (though schools are allowed to use the test more frequently). It may seem somewhat of a stretch to use a Grade 3 test result as the basis of a university exam dispensation. It may also lead to a higher proportion of students with a dyslexia diagnosis in upper and higher education than anticipated—as discussed below. It should be acknowledged that some students who had early difficulties learning to read may experience a successful remediation for a variety of reasons, such as intensive practice and many “protective factors” (van Viersen et al., 2019). It may not be helpful to maintain such children in a disabled identity, and special resources and provisions could be better spent on other children. Thus, an important topic for more (longitudinal) research is to contribute knowledge about the stability of a dyslexia diagnosis over time.

*Are L2 students properly served by the National Dyslexia Test?* Even though the National Dyslexia Test is relatively robust to vocabulary limitations, some knowledge of the dominant language (Danish) is required. The test instructions specify a threshold (below a score of 11) on the basic vocabulary test below which the Dyslexia Test score should be interpreted with caution. A dynamic measure of acquisition of word reading has been developed and implemented for adults (Elbro et al., 2012). This measure does not require knowledge of any particular language, and it is currently under standardization in school-age students.

*How much of a change does the National Dyslexia Test entail?* The strong overlap between the National Dyslexia Test results and previous classifications indicates that the definitions and practices did not differ substantially. The strong overlap is reassuring in terms of (external) convergent validity of the test. At the same time, it raises the question whether the National Dyslexia Test is really necessary. There are many perspectives to consider here, some of which were mentioned in “Introduction,” for example, that a shared operational definition promotes consensus and fairness across the educational system. The study added that the importance of a National Dyslexia Test may to some extent vary with educational level. For example, it appeared that post-compulsory education that attracts many poor readers is likely to be faced with a very complex task. Dyslexia is probably only one of a mixed range of disabilities that affect educational outcomes. This makes it important for many students that they receive the precise support that they need; neither schools nor the individual students have resources for mistakes. Another example is the high number of students in Grade 3 who receive some kind of special reading support. Perhaps, teachers and schools can use resources better with the help of the National Dyslexia Test.

### The National Dyslexia Test in practice

The National Dyslexia Test was made available to all schools and educational institutions in February 2015. Since the introduction, the Danish Ministry of Education has kept a record of each run of the test provided by the electronic system. The database is large and can serve several administrative and research purposes.

Two and a half years after its introduction, The National Dyslexia Test was in use in all 98 communes in Denmark (Danish Ministry of Education, 2017). By 2020, an average of 12% of all school-age students had taken the test out of whom 60% (7% of all) could be considered dyslexic (Danish Ministry of Education, 2020). Not surprisingly, the figure of 7% matches the 8% of students who received special service before the introduction of the National Dyslexia Test. The cut-off on the dyslexia scale was selected to provide this match. However, the cut-off did not take into account the cumulative effect of testing at different grade levels when a dyslexia test result is considered valid for 15 years. This can be seen in the fact that at the end of compulsory schooling, 16% of all students in Grade 9 (2021) had taken the test at least once, and about 69% of these students (or 11% of all) could be considered dyslexic (Danish Ministry of Education, 2021). This means that further education institutions will need to make provisions for more than the usual 8% students with dyslexia.

Access to special services now depends on a dyslexia diagnosis in which the National Dyslexia Test is a key indicator. Such services include provision of special teaching support, text-to-speech and speech-to-text support at exams, additional time at written exams and for written reports, and free teaching in small groups for adults.

Since its release, the National Dyslexia Test has become an anchor point for prediction and early intervention. For example, a newer national test of risk of dyslexia aimed at kindergarten and Grade 1 has been calibrated so as to provide a high prediction rate of dyslexia as indicated later by the National Dyslexia Test (Gellert & Elbro, 2016, 2018).

## Conclusion

It is an advantage for the majority of stakeholders to use a single dyslexia test across educational levels and institutions. It contributes fairness and transparency to have a common understanding of what is covered by the label “dyslexia” and where the borderlines are. This study indicates that a National Dyslexia Test can be developed so as to be reliable and valid from Grade 3 and all the way up to university levels. Practice attests to the usefulness of the test: although it is up to each of the 98 Danish municipalities to decide whether to use the test or not, they all use it. Hence, professionals appear to have accepted the test as a useful tool, even though cut-off points are, and should be, discussed. Possibly, research, too, would benefit from using, if not the same, then at least comparable tests across countries and research projects.

## Data Availability

The data that support the findings of this study are available from the corresponding author, MP, upon reasonable request.
