# Correction: A national test of dyslexia

**DOI:** 10.1007/s11881-026-00376-z

**Published:** 2026-05-22

**Authors:** Mads Poulsen, Holger Juul, Carsten Elbro

**Affiliations:** Department of Nordic Studies and Linguistics, University of Copenhagen, Emil Holms Kanal 2, Copenhagen S, 2300 Denmark

**Correction**: Annals of Dyslexia (2023) 73:337–355

**https://doi.org/10.1007/s11881-023-00285-5**

On this article, in Table 3. Some of the sensitivity and specificity values were calculated using incorrect threshold levels. These errors have been corrected with this erratum.

**Table:** 

Educational level	AUC [95% CI]	Sensitivity	Specificity
Compulsory schooling			
Grade 3	0.85 [0.77-0.92]	0.43	0.96
Grade 5	0.93 [0.89-0.97]	0.64	0.95
Grade 7	0.97 [0.94-0.99]	0.84	0.95
Grade 9	0.97 [0.95-0.99]	0.86	0.95
Upper secondary education			
Vocational training	0.76 [0.68-0.84]	0.61	0.80
Tech/commercial college	0.89 [0.82-0.95]	0.48	0.96
General college (A-levels)	0.95 [0.91-0.98]	0.79	0.98
Higher education			
Short higher ed. (1–2 y)	0.90 [0.82-0.97]	0.75	0.90
Medium higher ed. (3–4 y)	0.94 [0.90-0.98]	0.72	0.92
Long higher ed. (5 + y)	0.96 [0.93-0.99]	0.63	0.97

